# 

**Published:** 1823-03

**Authors:** 


### NOTICES TO CORRESPONDENTS.

*The Communications of various Correspondents are unavoidably postponed till next month, notwithstanding the omission of the* Collectanea, *and the addition of eight pages to the present Number. In the order of their insertion, we are guided as much as possible by the date of our receiving them. One paper in our last was dated* January, *instead of* November, *by mistake.*

*We shall feel much obliged to our Correspondent at Birmingham, if he will favour us with his name: we dislike anonymous Communications, particularly criticisms of other Correspondents.*

*The letter of* Dr. VALENTIN, *of Nancy, has been received. With respect to* Dr. JENNER, *we refer to the* Obituary *in the present Number: the other medical friend, about whom he inquires, has been dead for some time.*

*The Communication of* Dr. AMEDIE DUPAU *shall be attended to.*

*The letter of* TYRO *cannot, with propriety, be answered publicly: if he chooses to send us his address, we shall be happy to give him our best advice.*

*We beg to thank* Dr. BARON *for his polite attention in transmitting the particulars relative to his venerable friend* Dr. JENNER.

